# Functional long-term outcome following endovascular thrombectomy in patients with acute ischemic stroke

**DOI:** 10.1186/s42466-023-00301-4

**Published:** 2024-02-01

**Authors:** Andreas Rogalewski, Nele Klein, Anja Friedrich, Alkisti Kitsiou, Marie Schäbitz, Frédéric Zuhorn, Burkhard Gess, Björn Berger, Randolf Klingebiel, Wolf-Rüdiger Schäbitz

**Affiliations:** 1 Department of Neurology, Evangelisches Klinikum Bethel, University Hospital OWL of the University Bielefeld, Campus Bielefeld-Bethel, Schildescher Str. 99, 33611 Bielefeld, Germany; 2 Department of Neurology, Sankt Elisabeth Hospital Gütersloh, Catholic Hospital Association of East Westfalia (KHO), Gütersloh, Germany; 3 Department of Neurology, Medical Faculty, RWTH Aachen University, Aachen, Germany; 4 Department of Psychology, Bielefeld University, Bielefeld, Germany; 5 Department of Neuroradiology, Evangelisches Klinikum Bethel EvKB, University Hospital OWL of the University Bielefeld, Campus Bielefeld-Bethel, Bielefeld, Germany

**Keywords:** Endovascular treatment, Thrombectomy, Stroke, Large vessel occlusion, Long-term outcome, Modified Rankin Scale, Quality of life, HRQoL

## Abstract

Endovascular thrombectomy (EVT) is the most effective treatment for acute ischemic stroke caused by large vessel occlusion (LVO). Yet, long-term outcome (LTO) and health-related quality of life (HRQoL) in these patients have rarely been addressed, as opposed to modified Rankin scale (mRS) recordings. We analysed demographic data, treatment and neuroimaging parameters in 694 consecutive stroke patients in a maximum care hospital. In 138 of these patients with respect on receipt of written informed consent, LTO and HRQoL were collected over a period of 48 months after EVT using a standardised telephone survey (median 2.1 years after EVT). Age < 70 years (OR 4.82), lower NIHSS on admission (OR 1.11), NIHSS ≤ 10 after 24 h (OR 11.23) and complete recanalisation (mTICI3) (OR 7.79) were identified as independent predictors of favourable LTO. Occurrence of an infection requiring treatment within the first 72 h was recognised as a negative predictor for good long-term outcome (OR 0.22). Patients with mRS > 2 according to the telephone survey more often had complaints regarding mobility, self‐care, and usual activity domains of the HRQoL. Our results underline a sustainable positive effect of effective EVT on the quality of life in LVO stroke. Additionally, predictive parameters of outcome were identified, that may support clinical decision making in LVO stroke.

## Introduction

Endovascular thrombectomy (EVT) represents the standard medical treatment for patients with major vessel occlusion in acute ischemic stroke (AIS) in the anterior circulation. Its efficacy has been demonstrated by five randomized trials in 2015 [4, 8, 17, 23, 42]. This impressive success was attributed to stringent inclusion and exclusion criteria. EVT was shown to be effective in patients who presented with 1. Acute occlusion of the intracranial internal carotid artery or the first segment of the middle cerebral artery, 2. Acute moderate to severe symptoms, 3. A very good functional baseline, and 4. An early time window (< 6 h) for endovascular treatment. In this patient population, the “number needed to treat” by thrombectomy for improvement of functional outcome at 90 days (one point on the modified Rankin scale [mRS]) amounted to 2.6 [18]. In 2018, the DAWN and DEFUSE-3 trials showed a significantly better neurological outcome for EVT than standard drug therapy in selected patients with an extended time window of 6–24 h [1, 34]. Recently, EVT was shown to be similarly effective in basilar artery occlusion up to 24 h after stroke onset [24, 48]. The widespread use of EVT in clinical routine and an enlarging spectrum of indications, e.g. treatment of more distal vessel occlusions, and simplified imaging assessment (i.e. without multimodal imaging) urges the need for identifying predictors of complete recanalisation and good clinical outcome. The assessment of clinical outcome as well as outcome prediction usually are based on the mRS at day 90 [3, 10, 12, 26, 28, 31–33, 37, 38, 46, 47, 50–52], the standard outcome measure in AIS studies. Only a few studies have investigated factors influencing long-term outcome beyond day 90 [5, 14, 16, 36, 53].

Nowadays, outcome assessments after disease and treatment are transforming. Patient-reported outcome measures (PROMs) in neurological diseases came into focus and are a novel way to judge the patient outcome by self-rating, independent of physicians’ interpretation and evaluation. Interestingly, PROMs emerged as standard outcome measure in other neurological diseases such as myasthenia gravis, where they even defined the primary endpoint for evaluating the therapeutic efficacy of drugs in recent phase III trials [20, 21]. In AIS patients such an approach is not established, although importance and relevance of this outcome measure has been acknowledged [15, 25, 39]. Moreover, PROMs were reported to correlate with clinician and self-reported mRS and reliably represented the outcome after a mild stroke or transient ischemic attack at 90 days [35].

Therefore, assessment of long-term stroke outcome beyond 3 months as true indicator for quality of life and capacity of managing daily life activities of stroke patients is an increasingly relevant parameter to evaluate the treatment effect of EVT. Stroke long-term sequelae can affect physical and mental health and lead to reduced health-related quality of life (HRQoL) [30]. The HRQoL of people living with the sequelae of stroke has been reported to be lower than that of people living without the consequences of stroke [27]. The identification of factors influencing HRQoL provides an opportunity to develop effective strategies to improve HRQoL in stroke patients.

This study aimed at identifying predictors associated with good long-term outcome (> 90 day time interval) as well as HRQoL in patients receiving EVT.

## Methods

### Patient characteristics

We conducted an analysis of patient records from a major German hospital (tertiary referral centre, Evangelisches Klinikum Bethel, University Hospital OWL of the University Bielefeld). A total of 694 patients with acute ischemic stroke (AIS) and consecutive endovascular thrombectomy were identified between January 1st, 2017 and December 31st, 2020. Previous systemic thrombolysis was not considered an exclusion criterion.

All patients who received EVT were included, regardless of the affected vascular territory. The attending neurologist and interventional neuroradiologist consensually decided on a case-by-case basis whether or not endovascular treatment was indicated.

Furthermore, a long-term catamnesis was conducted via telephone. Written informed consent was required for participation in the telephone survey. Patients who declined to participate or did not return a signed consent form were not included in the analysis.

### Procedure

Patient characteristics were comprehensively assessed including demographics, aetiology of stroke, cardiovascular risk factors and neuroradiologic imaging findings (MR or computed tomography [CT] scans, echocardiography).

The patients’ initial blood pressure figures were recorded upon admission. Laboratory parameters were recorded on admission including blood glucose level, HbA1c, cholesterol level, LDL level, and CRP figures. Pre-existing statin therapy was recorded as well as pre-existing antiplatelet therapy or oral anticoagulation. Effective oral anticoagulation on admission was defined as INR ≥ 1.7 or anti-Xa ≥ 0.4 IU/ml or thrombin time ≥ 42 s. Stroke severity was assessed using the modified Rankin Scale and NIHSS.

Thrombectomy parameters comprised occluded target vessels, thrombectomy techniques applied, number of aspirations, need for carotid stent implantation, the side of infarction, and the degree of recanalisation success using the modified treatment in cerebral infarction (mTICI) score. A direct aspiration as first pass technique without need of repeated further aspiration was defined as first aspiration thrombectomy. Aspiration EVT only without usage of stent retriever was defined as primary aspiration alone, without the need of rescue stent retriever devices.

The recorded complication rates addressed intracranial haemorrhages (regardless of size and symptoms), symptomatic haemorrhages, epileptic seizures, recurrent strokes or transient ischemic attacks (TIA) within the first 72 h, infections requiring treatment within the first 72 h, and acute onset delirium.

The telephone interview was carried out by medical students who had been trained to conduct the interview using a standardised questionnaire. The telephone survey was conducted in a standardised manner by means of personal individual interviews with the patients or their relatives. The interviews started with the survey of the current mRS, the individual scales of the EQ-5D-5L by means of EQ-5D-5L Telephone interview version developed for the German language. Subsequently, questions with predefined answers were asked about the patient's living situation, pre-stroke care level and current care level, occupation, relapse events, renewed inpatient stay and current medication.

In the telephone interview, the mRS, the EQ-5D-5L questionnaire by the EuroQol Group as well as the EuroQol-visual analogue scale EQ-VAS were collected. The EQ-5D-5L questionnaire measures generic health-related quality of life, with the five dimensions (1) mobility, (2) self-care, (3) usual activities, (4) pain/discomfort, and (5) anxiety/depression. Each dimension is rated on a scale that describes the degree of problems in that area (i.e. no problems to walk, slight problems, moderate problems, severe problems, or unable to walk) with lower scores indicating a better HRQoL [7],“EuroQol–a New Facility for the Measurement of Health-Related Quality of Life,” 1990; [19, 22]. Furthermore, ongoing antiplatelet therapy, anticoagulation therapy, statin therapy, antihypertensive therapy, and antidepressant therapy were recorded.

### Data analysis

Data analysis was carried out using the Statistical Package for the Social Sciences (SPSS) version 25 (IBM, 2018). Descriptive statistics were displayed as mean ± standard deviation for continuous data and frequencies with percentages for categorical variables. Normal distribution of residuals was assessed via Shapiro–Wilk test with *p* < 0.05 indicating non-normal distribution. Homoscedasticity was assessed visually via q-q-plots.

In all calculations, a *p*-value of less than 0.05 in the two-sided test indicated statistical significance. Demographic characteristics were compared by using parametric t-tests or non-parametric Mann–Whitney U tests, depending on normal distribution. In order to identify potential predictors for a later binary logistic regression, preliminary analyses were carried out for the group differences of favourable (mRS 0–2) vs. poor outcome (mRS 3–6). Parametric t-tests or non-parametric Mann–Whitney U-tests were used for ordinal and interval dependent variables (e.g. blood pressure, serum glucose level, HbA1c); differences in categorical variables (e.g. hypertension, diabetes mellitus and hypercholesterolaemia) were assessed using chi-square tests. In a second step, all statistically significant variables from the preliminary analyses were entered into a linear regression model and tested for multicollinearity. Relevant multicollinearity was assumed when the variance inflation factor (VIF) was greater than 2. Removing individual predictors with high VIF ensured a model with low multicollinearity. Finally, we computed three binary logistic regression models with the enter method [ordinal variables were compared with the simple (first) method] in order to identify relevant predictors of good long-term functional outcome.

The HRQoL was analysed using the five dimensions, the visual analogue scale, and the index of the EQ-5D-5L questionnaire by the EuroQol Group. Logically, all dead patients (mRS = 6) had to be excluded, as they could not complete a telephone survey. Patients with a favourable outcome (mRS 0–2) were compared to patients with a poor outcome (mRS 3–5) regarding the five dimensions mobility, self-care, usual activities, pain/discomfort, and anxiety/depression, as well as the EuroQol visual analogue scale and the EQ-5D-5L index. In line with previous analyses, parametric t-tests or non-parametric Mann–Whitney U-tests were performed depending on distribution characteristics.

In order to correct for alpha error accumulation in multiple testing, *p*-values were adjusted separately for each set of analyses (favourable outcome, HRQoL, mortality, recanalisation) using the Bonferroni method (p_adj_ = p_obs_*k; where p_adj_: adjusted *p*-value, p_obs_: observed *p*-value, k = number of comparisons) [6].

## Results

### Demographic characteristics

In total, 694 patients were included, regardless of the affected vascular territory. Mean age was 74.0 ± 13.4 years (Minimum: 18, Maximum: 104, Median: 77 years). The sex distribution showed no difference (Females: 52.0%). 134 patients (19.3%) died during hospitalisation (mRS at discharge score 6).

Of the remaining 560 patients, 138 patients or their relatives gave written informed consent to participate in a telephone survey on long-term follow-up. These 138 patients were included in the long-term follow-up analysis. The mean follow-up interval was 2.2 ± 1.2 years (median: 2.1 years).

Baseline demographic and clinical data, MRI parameters as well as details of EVT of both groups (whole population of 694 patients and sample of the telephone survey with 138 patients) are summarised in Table 1. The comparison of the two groups showed that the telephone survey sample was younger than the overall population (70.8 ± 14.7 years vs. 74.0 ± 13.4 years, *p* = 0.018) and had a lower mRS at discharge (3.1 ± 1.6 vs. 3.7 ± 1.7, *p* = 0.001). The difference in mRS was mainly caused by a higher rate of deceased patients at discharge in the overall population compared to the proportion of deceased patients in the long-term follow-up (19.8% vs. 8.0%, *p* < 0.001). The MRI findings were compared in 15.4% of all patients and 21.7% of patients with long-term follow-up. Here, the patients in the long-term course showed a lower extent of cerebral microangiopathy measured using Fazekas’ score. The other parameters showed no significant differences between the two groups (see Table 1). Results on mRS, EQindex, care delivery, and medication of patients in the telephone survey are also shown in Table 1.

**Table 1:** Demographic characteristic, risk factors and thrombectomy parameters of the whole study population (N = 694) and the sample of the telephone survey (N = 138)

	Whole study population (N = 694)	Sample of telephone survey (N = 138)	Test statistics
Age	74.0 ± 13.4	70.8 ± 14.7	**U = 41803.000, Z = 2.360, *p* = 0.018**^**c**^
Sex—Female	361/694 (52.0%)	71/138 (51.4%)	0.015/0.903^a^
Hypertension	609/694 (87.8%)	120/138 (87.0%)	0.067/0.795^a^
Diabetes mellitus	130/694 (18.7%)	23/138 (16.7%)	0.327/0.567^a^
Atrial fibrillation	330/694 (47.6%)	65/138 (47.1%)	0.009/0.923^a^
Former stroke	188/694 (27.1%)	35/138 (25.4%)	0.175/0.676^a^
Antiaggregant therapy on admission	187/694 (26.9%)	42/138 (30.4%)	0.665/0.415^a^
Oral anticoagulation on admission (INR > = 1.7) or (AntiXa > = 0.4 IU/ml) or (thrombin time > = 42 s)	41/694 (5.9%)	10/138 (7.2%)	0.358/0.549^a^
Statin therapy on admission	162/694 (23.3%)	34/138 (24.6%)	0.125/0.724^a^
Aetiology of stroke			
LAAS	107 (15.4%)	18 (13.0%)	4.344/0.227^a^
CES	302 (43.5%)	66 (47.8%)	
Other	29 (4.2%)	11 (8.0%)	
Cryptogenic stroke	177 (25.5%)	32 (23.2%)	
Missing	79 (11.4%)	11 (8.0%)	
Event to door time			
< 1 h	75/694 (10.8%)	17/138 (12.3%)	
1–2 h	134/694 (19.3%)	31/138 (22.5%)	
2–3 h	109/694 (15.7%)	20/138 (14.5%)	
3–4 h	72/694 (10.4%)	14/138 (10.1%)	
4–5 h	44/694 (6.3%)	10/138 (7.2%)	
5–6 h	41/694 (5.9%)	3/138 (2.2%)	
6–24 h	80/694 (11.5%)	18/138 (13.0%)	
In house-stroke	33/694 (4.7%)	8/138 (5.8%)	
Unknown	95/694 (13.7%)	17/138 (12.3%)	5.903/0.750^a^
Intravenous thrombolysis	235/694 (33.9%)	55/138 (39.9%)	1.821/0.177^a^
Door to needle time	40.8 ± 20.3 min	41.8 ± 20.8 min	U = 6736.500, Z = 0.490, *p* = 0.624^c^
	(Median 36)	(Median 38)	
Door to groin time	98.4 ± 78.2 min	97.3 ± 62.1 min	U = 37307.500, Z = 0.084, *p* = 0.933^c^
	Median: 84	Median: 85	
Local intra-arterial thrombolysis	8/694 (1.2%)	3/138 (2.2%)	–^d^
Severity indices on admission			
Modified Rankin Scale on admission	4.4 ± 0.9	4.2 ± 1.0	U = 39685.000, Z = 1.706, *p* = 0.088^c^
	Median: 5	Median: 5	
Modified Rankin Scale on admission	Score 0: 2 (0.3%)	Score 0: 1 (0.7%)	8.857/0.115^a^
	Score 1: 3 (0.4%)	Score 1: 0 (0%)	
	Score 2: 22 (3.2%)	Score 2: 4 (2.9%)	
	Score 3: 84 (12.1%)	Score 3: 28 (20.3%)	
	Score 4: 149 (21.5%)	Score 4: 25 (18.1%)	
	Score 5: 411 (59.2%)	Score 5: 71 (51.4%)	
	Missing: 23 (3.3%)	Missing: 9 (6.5%)	
Modified Rankin Scale at discharge	Score 0: 20 (2.9%)	Score 0: 5 (3.7%)	**13.971/0.030**^**a**^
	Score 1: 57 (8.4%)	Score 1: 19 (14.2%)	
	Score 2: 126 (18.6%)	Score 2: 28 (20.9%)	
	Score 3: 121 (17.8%)	Score 3: 29 (21.6%)	
	Score 4: 102 (15.0%)	Score 4: 18 (13.4%)	
	Score 5: 118 (17.4%)	Score 5: 24 (17.9%)	
	Score 6: 134 (19.8%)	Score 6: 11 (8.2%)	
Modified Rankin Scale at discharge	3.7 ± 1.7	3.1 ± 1.6	**U = 37644.000, Z = 3.182, *p* = 0.001**^**c**^
	Median: 4	Median: 3	
NIHSS on admission	15.6 ± 8.3	14.7 ± 7.3	U = 41300.500, Z = 1.212, *p* = 0.225^c^
	Median: 15	Median: 15	
NIHSS after 24 h	11.1 ± 8.2	9.9 ± 8.6	U = 24634.500, Z = 1.726, *p* = 0.084^c^
	Median: 9	Median: 7	
NIHSS at discharge	6.1 ± 10.8	13.3 ± 19.4	U = 33000.000, Z = 0.539, *p* = 0.645^c^
	Median: 3.5	Median: 5.5	
Thrombectomy parameters			
Occluded target vessel			
M1	445/694 (64.1%)	89/138 (64.5%)	0.007/0.934^a^
M2	242/694 (34.9%)	48/138 (34.8%)	0.000/0.984^a^
M3	28/694 (4.0%)	6/138 (4.3%)	0.029/0.865^a^
M4	1/694 (0.1%)	1/138 (0.7%)	–^d^
ICA	176/694 (25.4%)	41/138 (29.7%)	1.130/0.288^a^
Basilar artery	61/694 (8.8%)	9/138 (6.5%)	0.768/0.381^a^
VA	12/694 (1.7%)	1/138 (0.7%)	–^d^
ACA	104/694 (15.0%)	19/138 (13.8%)	0.135/0.713^a^
PCA	38/694 (5.5%)	7/138 (5.1%)	0.037/0.848^a^
ACC	8/694 (1.2%)	1/138 (0.7%)	–^d^
SUCA	6/694 (0.9%)	2/138 (1.4%)	–^d^
Thrombectomy technique			
Single (vs. multiple) aspirations	308/694 (44.4%)	59/138 (42.8%)	0.352/0.553^a^
Stent retriever	435/694 (62.7%)	80/138 (58.0%)	1419/0.234^a^
Angioplasty	10/694 (1.4%)	3/138 (2.2%)	–^d^
Stentimplantation	116/694 (16.7%)	21/138 (15.2%)	0.199/0.655^a^
Result of thrombectomy			
TICI3	423/694 (61.0%)	91/138 (65.9%)	1.173/0.279^a^
TICI2b or TICI3	618/694 (89.0%)	128/138 (92.8%)	1.705/0.192^a^
Complications			
Intracerebral bleeding	300/694 (43.2%)	56/138 (40.6%)	0.286/0.593^a^
Symptomatic bleeding	18/694 (2.6%)	1/138 (0.7%)	–^d^
Seizure	34/694 (4.9%)	4/138 (2.9%)	–^d^
Recurrent stroke/TIA within 72 h	6/694 (0.9%)	0/138 (0%)	–^d^
Infection within 72 h	355/694 (51.2%)	71/138 (51.4%)	0.004/0.949^a^
Delirium	35/694 (5.0%)	8/138 (5.8%)	0.133/0.715^a^
MRI parameters			
MRI performed	107/694 (15.4%)	30/138 (21.7%)	3.344/0.067^a^
Volume of infarction	49.2 ± 56.7 ml	48.5 ± 48.7 ml	U = 1169.500, Z = 0.277, *p* = 0.781^c^
Degree of cerebral microangiopathy (Fazekas’ Score)	0: 20/102 (19.6%)	0: 11/29 (37.9%)	**U = 1119.500, Z = 2.100, *p* = 0.036**^**c**^
	1: 43/102 (42.2%)	1: 12/29 (41.4%)	
	2: 25/102 (24.5%)	2: 3/29 (10.3%)	
	3: 14/102 (14.7%)	3: 3/29 (10.3%)	
Fragmented infarction	91/103 (88.3%)	25/28 (89.3%)	0.019/0.890^a^
Fragmented infarction	Singular: 13/99 (13.1%)	Singular: 3/25 (12.0%)	U = 1225.500, Z = 0.078, *p* = 0.938^c^
	≥ 1 and < 5: 28/99 (28.3%)	≥ 1 and < 5: 9/25 (36.0%)	
	5—< 10: 26/99 (26.3%)	5—< 10: 4/25 (16.0%)	
	≥ 10: 32/99 (32.3%)	≥ 10: 9/25 (36.0%)	
Multi territorial infarction	59/103 (57.3%)	12/29 (41.4%)	2.302/0.129^a^
Brainstem or cerebellum involvement	45/103 (43.7%)	8/28 (28.6%)	2.089/0.148^a^
Involvement posterior circulation area	56/104 (53.8%)	11/29 (37.9%)	2.298/0.130^a^
Involvement anterior circulation area	67/104 (64.4%)	22/29 (75.9%)	1.340/0.247^a^
Telephone survey			
Interval after stroke		2.2 ± 1.2 years	
		(Median: 2.1 years)	
Modified Rankin Scale on admission		Score 0: 15 (10.9%)	
		Score 1: 23 (16.7%)	
		Score 2: 22 (15.9%)	
		Score 3: 19 (13.8%)	
		Score 4: 17 (12.3%)	
		Score 5: 7 (5.1%)	
		Missing: 35 (25.4%)	
Mobility		2.6 ± 1.3 (Median: 3)	
Self-care		2.3 ± 1.5 (Median: 2)	
Usual activities		2.7 ± 1.5 (Median: 3)	
Pain/discomfort		2.0 ± 1.1 (Median: 2)	
Anxiety/depression		2.4 ± 1.1 (Median: 2)	
EuroQol-visual analogue scales (EQ-VAS)		55.1 ± 25.2	
German EQ-5D-5L index		0.635 ± 0.318	
German level of care		0: 49/102 (48.0%)	
		1: 5/102 (4.9%)	
		2: 15/102 (14.7%)	
		3: 14 (13.7%)	
		4: 11 (10.8%)	
		5: 8 (7.8%)	
		Missing: 36	
Ongoing antiaggregant therapy		47/101 (46.5%)	
Ongoing oral anticoagulation		50/102 (49.0%)	
Ongoing statin therapy		75/101 (74.3%)	
Ongoing antihypertensive therapy		83/101 (82.2%)	
Ongoing antidepressive therapy		19/101 (18.8%)	

^a^Chi square, ^b^parametric t-test, and ^c^Mann-Whitney U-Test used as appropriate. ^d^Categorical variables with cell frequencies lower than 5 were not analysed due to requirement violations of the Chi-square tests

Parameters highlighted in bold indicate significant differences between the groups

### Predictors associated with favourable long-term outcome

Favourable long-term outcome was defined as a mRS score of 0 to 2 at the time of the telephone survey. 60 out of 138 patients had a mRS between 0 and 2 in the telephone survey (43.5%). Subsequently, comparisons were made between these 60 patients and those with mRS scores between 3 and 6 (n = 78). 35 out of 138 patients (25.4%) had a mRS score 6 at telephone survey.

**Fig. 1:**
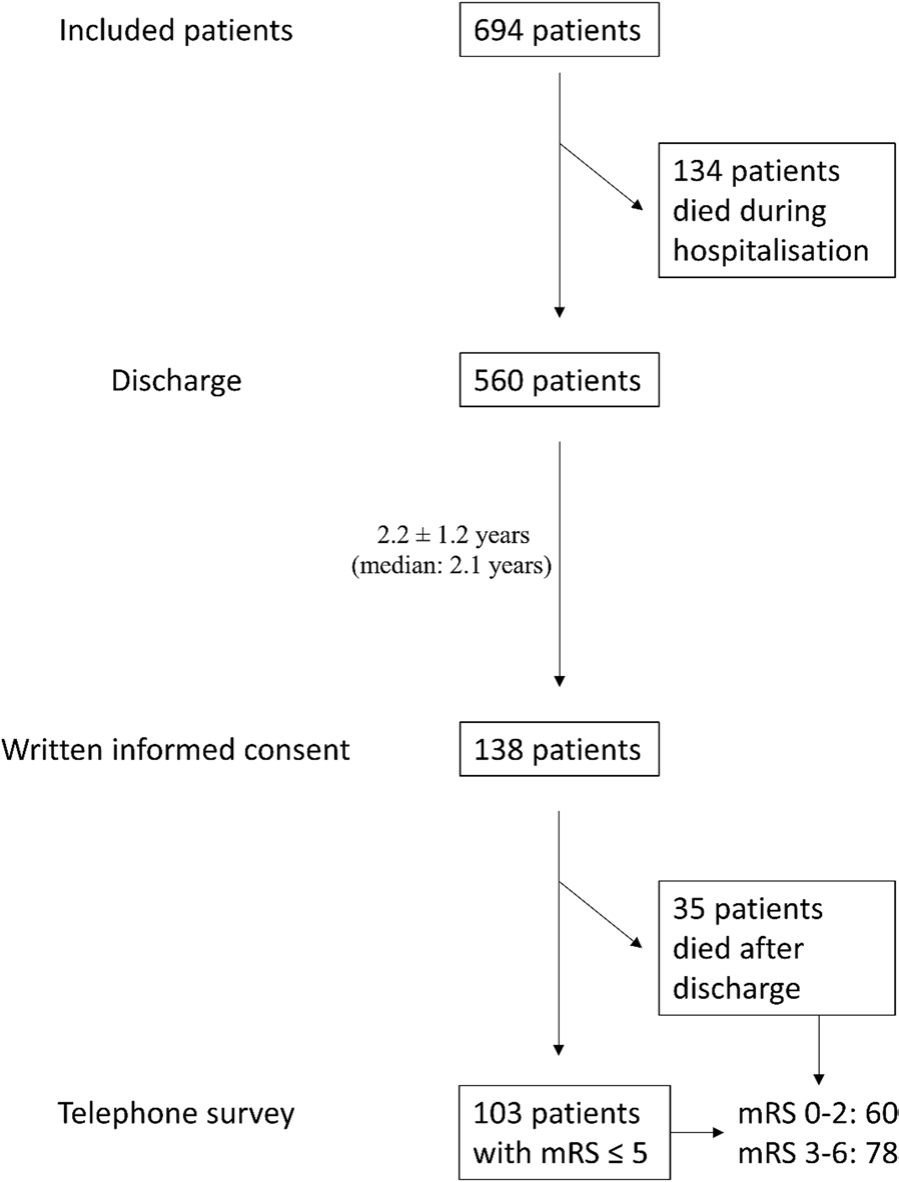
Presentation of the temporal order from inclusion of patients (N = 694), discharge (N = 560), written informed consent (N = 138) to inclusion in the telephone survey (N = 103) is presented. It should be noted that the analysis of long-term follow-up by mRS included 138 patients inclusive of those who died in the meantime (mRS = 6), whereas the analysis of HRQoL included 103 patients without 35 patients with mRS = 6

Taking into account the 134 patients who had already died during hospitalisation, a total of 169 patients died during the follow-up period, i.e. 169 out of 694 patients of the total collective (24.4%) or 169 out of 257 patients whose status was reported in the long-term follow-up (65.8%). The temporal order of patients from inclusion in the study, written informed consent, and inclusion in the long-term outcome analysis is shown in Fig. 1.

Patients with favourable long-term outcome were younger (67.0 ± 15.5 years vs. 73.7 ± 13.5, *p* = 0.004), were less severely affected on admission (NIHSS: 11.5 ± 5.5 vs. 17.1 ± 7.6, *p* < 0.001) as well as 24 h after admission post EVT (NIHSS: 5.2 ± 5.4 vs. 14.8 ± 8.6, *p* < 0.001). Carotid stent implantation was required more often (23.3% vs. 9.0%; *p* = 0.020), and these patients had more frequent complete recanalisation (mTICI 3: 76.7% vs. 57.7%, *p* = 0.020). Favourable long-term outcome was negative associated with atrial fibrillation (36.7% vs. 55.1%; *p* = 0.031) as well as with occurrence of infections requiring treatment within the first 72 h (36.7% vs. 62.8%, *p* = 0.002).

Further parameters depending on the outcome are shown in Table 2. Based on previous study results, age was dichotomized with a cut off value < 70 years [3, 10], NIHSS at 24 h with a cut off value ≤ 10 [50].

**Table 2:** Predictors associated with good long-term outcome after undergoing mechanical thrombectomy assessed by telephone and dichotomized mRS (mRS = 0–2 versus greater than or equal to 3)

	mRS 0–2 (N = 60)	mRS ≥ 3 (N = 78)	Test statistics
Age	67.0 ± 15.5	73.7 ± 13.5	**U = 3007.000, Z = 2.867, *p* = 0.004**^**c**^
Sex—Female	28/60 (46.7%)	43/78 (55.1%)	0.972/0.324^a^
Aetiology of stroke			
LAAS	10 (16.9%)	8 (11.8%)	
CES	27 (45.8%)	39 (57.4%)	
Other	5 (8.5%)	6 (8.8%)	
Cryptogenic stroke	17 (28.8%)	15 (22.1%)	1.992/0.574^a^
Event to door time			
≤ 2 h	21/52 (40.4%)	27/61 (44.3%)	0.173/0.678^a^
Door to needle time	37.1 ± 18.5 min	47.1 ± 22.2 min	U = 478.500,
	(Median 35)	(Median 40.5)	Z = 1.713, *p* = 0.089^c^
Door to groin time	92.0 ± 43.7 min	101.2 ± 73.0 min	U = 1730.000, Z = 0.021, *p* = 0.983^c^
	Median: 86	Median: 81.5	
Intravenous thrombolysis	29/60 (48.3%)	26/78 (33.3%)	3.183/0.074^a^
Local intra-arterial thrombolysis	2/60 (3.3%)	1/78 (1.3%)	–^d^
Severity indices on admission			
Modified Rankin Scale on admission	4.0 ± 1.1	4.4 ± 0.9	U = 2397.000, Z = 1.909, *p* = 0.056^c^
	Median: 4.0	Median: 5	
Modified Rankin Scale on admission	Score 0: 1 (1.8%)	Score 0: 0 (0%)	5.720/0.221^a^
	Score 1: 0 (0%)	Score 1: 0 (0%)	
	Score 2: 3 (5.5%)	Score 2: 1 (1.4%)	
	Score 3: 13 (23.6%)	Score 3: 15 (20.3%)	
	Score 4: 13 (23.6%)	Score 4: 12 (16.2%)	
	Score 5: 25 (45.5%)	Score 5: 46 (62.2%)	
NIHSS on admission	11.5 ± 5.5	17.1 ± 7.6	**U = 2997.500, Z = 4.360, *p* < 0.001**^**c**^
	Median: 12.5	Median: 16.5	
NIHSS after 24 h	5.2 ± 5.4	14.8 ± 8.6	**U = 2481.500, Z = 6.305, *p* < 0.001**^**c**^
	Median: 3	Median: 14	
Delta NIHSS baseline to 24 h ≥ 8	21/51 (41.2%)	12/51 (23.5%)	3.628/0.057^a^
NIHSS ≤ 10 after 24 h	48/55 (87.3%)	18/53 (34.0%)	**32.277 / < 0.001**^**a**^
Thrombectomy parameters			
Occluded target vessel			
M1	36/60 (60.0%)	53/78 (67.9%)	0.936/0.333^a^
M2	21/60 (35.0%)	27/78 (34.6%)	0.002/0.962^a^
M3	4/60 (6.7%)	2/78 (2.6%)	–^d^
ICA	16/60 (26.7%)	25/78 (32.1%)	0.471/0.493^a^
Basilar artery	3/60 (5.0%)	6/78 (7.7%)	–^d^
VA	1/60 (1.7%)	0/78 (0%)	–^d^
ACA	5/60 (8.3%)	14/78 (17.9%)	2.641/0.104^a^
PCA	3/60 (5.0%)	4/78 (5.1%)	–^d^
ACC	0/60 (0%)	1/78 (1.3%)	–^d^
SUCA	0/60 (0%)	2/78 (2.6%)	–^d^
ACP	0/60 (0%)	1/78 (1.3%)	–^d^
Thrombectomy technique			
Single (vs. multiple) aspirations	25/60 (41.7%)	34/78 (44.2%)	0.085/0.770^a^
Stent retriever	33/60 (55.0%)	47/78 (61.0%)	0.506/0.477^a^
Angioplasty	3/60 (5.0%)	0/78 (0%)	–^d^
Stentimplantation	14/60 (23.3%)	7/78 (9.0%)	**5.420**/**0.020**^**a**^
Result of thrombectomy			
TICI3	46/60 (76.7%)	45/78 (57.7%)	**5.437**/**0.020**^**a**^
TICI2b or TICI3	58/60 (96.7%)	70/78 (89.7%)	2.418/0.120^a^
Risk factors			
Hypertension	49/60 (81.7%)	71/78 (91.0%)	2.619/0.106^a^
Systolic blood pressure on admission	154.7 ± 27.8 mm Hg	153.4 ± 26.3 mm Hg	U = 2023.000, Z = 0.100, *p* = 0.920^c^
Diastolic blood pressure on admission	85.3 ± 15.0 mm Hg	86.6 ± 19.0 mm Hg	U = 1998.500, Z = 0.084, *p* = 0.933^c^
Diabetes mellitus	10/60 (16.7%)	13/78 (16.7%)	0.000/1.000^a^
Blood glucose level on admission	130.8 ± 53.0 mg/dl	130.3 ± 31.2 mg/dl	U = 2492.500, Z = 1.255, p = 0.209^c^
HbA1c	40.6 ± 11.9 mmol/mol	40.4 ± 7.7 mmol/mol	U = 2400.000, Z = 0.950, *p* = 0.342^c^
Cholesterol level on admission	174.6 ± 45.2 mg/dl	174.1 ± 42.7 mg/dl	U = 2157.500, Z = 0.280, *p* = 0.780^c^
LDL level on admission	110.2 ± 42.3 mg/dl	113.1 ± 40.4 mg/dl	U = 2318.000, Z = 0.439, *p* = 0.661^c^
Statine therapy on admission	11/44 (25.0%)	23/63 (36.5%)	1.583/0.208^a^
CRP level on admission	12.6 ± 28.6 mg/dl	13.1 ± 22.9 mg/dl	U = 2592.000, Z = 1.596, *p* = 0.110^c^
Atrial fibrillation	22/60 (36.7%)	43/78 (55.1%)	**4.639**/**0.031**^**a**^
Former stroke	14/60 (23.3%)	21/78 (26.9%)	0.231/0.631^a^
Antiaggregant therapy on admission	18/46 (39.1%)	24/64 (37.5%)	0.030/0.862^a^
Oral anticoagulation on admission	4/60 (6.7%)	6/78 (7.7%)	–^d^
(INR > = 1.7) or (AntiXa > = 0.4 IU/ml) or (thrombin time > = 42 s)			
Cardial diagnostics			
Regional wall motion abnormality in TTE	3/39 (7.7%)	13/55 (23.6%)	–^d^
PFO in TEE	10/29 (34.5%)	6/29 (20.7%)	1.381/0.240^a^
Aortal plaques in TEE	25/27 (92.6%)	21/26 (80.8%)	1.615/0.204^a^
Other abnormalities in TEE	13/29 (44.8%)	6/28 (21.4%)	3.510/0.061^a^
Complications			
Intracerebral bleeding	19/60 (31.7%)	37/77 (48.1%)	3.746/0.053^a^
Symptomatic bleeding	0/60 (0%)	1/78 (1.3%)	–^d^
Seizure	1/60 (1.7%)	3/78 (3.8%)	–^d^
Recurrent stroke/TIA within 72 h	0/60 (0%)	0/78 (0%)	–^d^
Recurrent stroke after discharge	5/59 (8.5%)	4/43 (9.3%)	–^d^
Infection within 72 h	22/60 (36.7%)	49/78 (62.8%)	**9.287**/**0.002**^**a**^
Delirium	3/60 (5.0%)	5/78 (6.4%)	–^d^
MRI parameters			
Volume of infarction	42.1 ± 37.8 ml	61.4 ± 66.8 ml	U = 66.000, Z = 0.903, *p* = 0.928^c^
Degree of cerebral microangiopathy (Fazekas’ Score)	0: 8/16 (50.0%)	0: 3/13 (23.1%)	U = 138.000, Z = 0.111, *p* = 0.144^c^
	1: 6/16 (37.5%)	1: 6/13 (46.2%)	
	2: 1/16 (6.3%)	2: 2/13 (15.4%)	
	3: 1/16 (6.3%)	3: 2/13 (15.4%)	
Fragmented infarction	15/16 (93.8%)	10/12 (83.3%)	0.778/0.378^a^
Fragmented infarction	Singular: 1/16 (6.3%)	Singular: 2/9 (22.2%)	U = 47.500, Z = 0.144, *p* = 0.169^c^
	≥ 1 and < 5: 5/16 (31.3%)	≥ 1 and < 5: 4/9 (44.4%)	
	5—< 10: 3/16 (18.8%)	5—< 10: 1/9 (11.1%)	
	≥ 10: 7/16 (43.8%)	≥ 10: 2/9 (22.2%)	
Multi territorial infarction	7/17 (41.2%)	5/12 (41.7%)	0.001/0.979^a^
Brainstem or cerebellum involvement	5/17 (29.4%)	3/11 (27.3%)	–^d^
Involvement posterior circulation area	6/17 (35.3%)	5/12 (41.7%)	0.121/0.728^a^
Involvement anterior circulation area	13/17 (76.5%)	9/12 (75.0%)	0.008/0.927^a^
Recent therapy			
Antiaggregant therapy at telephone interview	29/58 (50.0%)	18/43 (41.9%)	0.658/0.417^a^
Oral anticoagulation at telephone interview	26/59 (44.1%)	24/43 (55.8%)	1.373/0.241^a^
Statine therapy at telephone interview	46/58 (79.3%)	29/43 (67.4%)	1.820/0.177^a^
Antihypertensive therapy at telephone interview	43/58 (74.1%)	40/43 (93.0%)	**6.013**/**0.014**^**a**^
Antidepressive therapy at telephone interview	9/58 (15.5%)	10/43 (23.3%)	0.968/0.325^a^
EQ-5D-5L (HRQoL)	mRS 0–2 (n = 60)	mRS 3–5 (n = 43)	
Mobility	1: 45.0%	1: 2.3%	**U = 2194.500, Z = 6.221, *p* < 0.001**
	2: 28.3%	2: 14.0%	
	3: 20.0%	3: 37.2%	
	4: 5.0%	4: 25.6%	
	5: 1.7%	5: 20.9%	
Self-care	1: 73.3%	1: 16.3%	**U = 2187.000, Z = 6.431, *p* < 0.001**
	2: 16.7%	2: 11.6%	
	3: 5.0%	3: 16.3%	
	4: 1.7%	4: 25.6%	
	5: 3.3%	5: 30.2%	
Usual activities	1: 51.7%	1: 2.3%	**U = 2234.500, Z = 6.482, *p* < 0.001**
	2: 23.3%	2: 9.3%	
	3: 10.0%	3: 25.6%	
	4: 11.7%	4: 27.9%	
	5: 3.3%	5: 34.9%	
Pain/discomfort	1: 46.7%	1: 41.9%	U = 1393.000, Z = 0.731, *p* = 0.465
	2: 20.0%	2: 16.3%	
	3: 23.3%	3: 27.9%	
	4: 8.3%	4: 14.0%	
	5: 1.7%	5: 0%	
Anxiety/depression	1: 36.7%	1: 18.6%	U = 1530.000, Z = 1.661, *p* = 0.097
	2: 20.0%	2: 30.2%	
	3: 28.3%	3: 25.6%	
	4: 15.0%	4: 25.6%	
	5: 0%	5: 0%	

^a^Chi square, ^b^parametric t-test, and ^c^Mann-Whitney U-Test used as appropriate. ^d^Categorical variables with cell frequencies lower than 5 were not analysed due to requirement violations of the Chi-square tests

Parameters highlighted in bold indicate significant differences between the groups

### Testing for multicollinearity and binary logistic regression for favourable long-term outcome

The variance inflation factor was less than 1.500 for all variables. Predictors of good long-term outcome in binary logistic regression analysis comprised age below 70 (OR 4.85 [95% CI 1.10–21.28], *p* = 0.037), NIHSS on admission (OR 1.11 [95% CI 1.002–1.23], *p* = 0.046), NIHSS 24 h post EVT ≤ 10 (OR 11.23 [95% CI 2.94–42.94], *p* < 0.001), and complete recanalisation mTICI 3 after EVT (OR 7.79 [95% CI 1.67–36.35], *p* = 0.009). Occurrence of infections requiring treatment within the first 72 h was a negative predictor for good long-term outcome (OR 0.22 [95% CI 0.06–0.84], *p* = 0.027).

The logistic regression model showed good predictive power (Cox & Snell R^2^: 0.455; Nagelkerkes R^2^: 0.607; Hosmer–Lemeshow-Test: Chi-Quadrat 9.017, *p* = 0.341).

### Health-related quality of life assessment using EQ-5D-5L

**Fig. 2:**
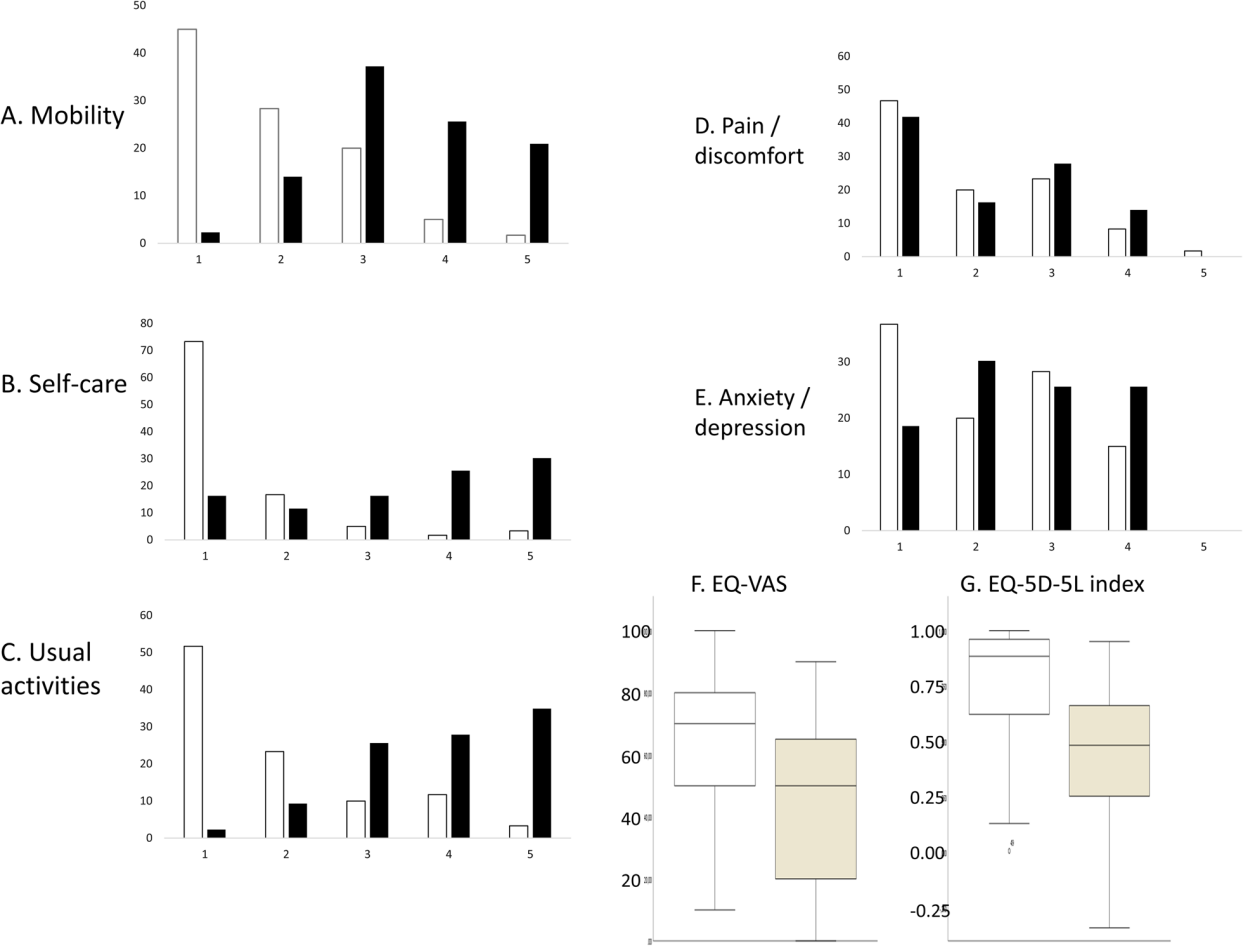
The patients' health-related quality of life (HRQoL) was assessed via telephone interview using the generic health-related quality of life instrument, the EQ-5D-5L questionnaire by the EuroQol Group, with the five dimensions: mobility, self-care, usual activities, pain/discomfort, and anxiety/depression. Each dimension is rated on five-point scale, ranging from “no problem (1)” to “extreme problems (5)”. The figure shows the distribution of responses for each domain (**A**–**E**) and box plots for the EuroQol-visual analogue scales (EQ-VAS) and the German EQ-5D-5L index for patients with favourable long-term outcome (mRS 0–2, white bars) vs. unfavourable long-term outcome (mRS 3–5, A-E: black bars, F + G: brown bars)

An overview over the five dimensions of the EQ-5D-5L questionnaire, the EuroQol-visual analogue scales (EQ-VAS) and the German EQ-5D-5L index is presented for favourable or unfavourable long-term outcomes in Fig. 2. More detailed data are provided in Table 2.

Significant differences were found in the following dimensions between patients with mRS 0–2 and the surviving interviewed patients with mRS 3–5: Patients with favourable long-term outcome showed better mobility (Mann–Whitney-U = 2194.500, Z = 6.431, *p* < 0.001), better self-care (Mann–Whitney-U = 2187.000, Z = 6.431, *p* < 0.001), and less problems with usual activities (Mann–Whitney-U = 2234.500, Z = 6.482, *p* < 0.001). The dimensions pain/discomfort as well as anxiety/depression revealed no significant differences. The EuroQol-visual analogue scales (EQ-VAS) was also higher in patients with mRS 0–2 than in the comparison group (63.5 ± 21.3 vs. 43.7 ± 25.7, U = 642.000, Z = 3.625, *p* < 0.001), the same applied to the German EQ-5D-5L index (0.77 ± 0.25 vs. 0.45 ± 0.31, U = 510.500, Z = 5.215, *p* < 0.001) [29]. This indicates that a positive long-term outcome is closely related to a better HRQoL.

Analyses of individual possible predictors of good long-term outcome measured in each individual dimension of the EQ-5D-5L scale at the time of the telephone survey were also conducted. In each case, a score of 1 or 2 was assumed to be good and compared with a score of 3–5. Possible predictors with significant differences were listed in Table 3 (activity dimension), Table 4 (self-care), Table 5 (usual activity), Table 6 (pain/discomfort), and Table 7 (anxiety). The results can be found in Table 3, 4, 5, 6 and 7.

**Table 3:** Predictors associated with good long-term outcome after undergoing mechanical thrombectomy assessed by telephone and dichotomized EQ mobility score by the EQ-5D-5L (score 1 or 2 versus greater than or equal to 3)

	Mobility score EQ-5D-5L 1 or 2 (N = 51)	Mobility score EQ-5D-5L 3–5 (N = 52)	Test statistics
Age	63.4 ± 14.9	71.9 ± 14.5	**U = 1841.000, Z = 3.399, *p* < 0.001**^**c**^
Aetiology of stroke			
LAAS	9 (18.0%)	4 (8.2%)	
CES	18 (36.0%)	31 (63.3%)	
Other	5 (10.0%)	5 (10.2%)	
Cryptogenic stroke	18 (36.0%)	9 (18.4%)	**8.363**/**0.039**^**a**^
CES	18/50 (36.0%)	31/49 (63.3%)	**7.360**/**0.007**^**a**^
Severity indices on admission			
NIHSS after 24 h	5.7 ± 5.6	10.8 ± 7.4	**U = 1273.500, Z = 3.561, *p* < 0.001**^**c**^
	Median: 4	Median: 8	
Delta NIHSS baseline to 24 h ≥ 8	20/42 (47.6%)	7/37 (18.9%)	**7.202**/**0.007**^**a**^
NIHSS ≤ 10 after 24 h	37/45 (82.2%)	22/39 (56.4%)	**6.659**/**0.010**^**a**^
Thrombectomy parameters			
Thrombectomy technique			
Stentimplantation	13/51 (25.5%)	4/52 (7.7%)	**5.918**/**0.015**^**a**^
Risk factors			
Blood glucose level on admission	130.6 ± 56.3 mg/dl	133.4 ± 28.3 mg/dl	**U = 1535.000, Z = 1.969, *p* = 0.049**^**c**^
Cholesterol level on admission	184.8 ± 43.8 mg/dl	164.9 ± 35.6 mg/dl	**U = 907.000, Z = 2.365, *p* = 0.018**^**c**^
LDL level on admission	121.4 ± 42.1 mg/dl	102.6 ± 32.1 mg/dl	**U = 934.000, Z = 2.179, *p* = 0.029**^**c**^
Statine therapy on admission	7/37 (18.9%)	17/39 (43.6%)	**5.349**/**0.021**^**a**^
Atrial fibrillation	13/51 (25.5%)	31/52 (59.6%)	**12.253 / < 0.001**^**a**^
Former stroke	6/51 (11.8%)	16/52 (30.8%)	**5.536**/**0.019**^**a**^
Oral anticoagulation on admission	1/51 (2.0%)	8/52 (15.4%)	**5.818**/**0.016**^**a**^
(INR > = 1.7) or (AntiXa > = 0.4 IU/ml) or (thrombin time > = 42 s)			
Complications			
Infection within 72 h	18/51 (35.3%)	35/52 (67.3%)	**10.564**/**0.001**^**a**^
Recent therapy			
Antiaggregant therapy at telephone interview	29/50 (58.0%)	18/51 (35.3%)	**5.232**/**0.022**^**a**^
Oral anticoagulation at telephone interview	18/51 (35.3%)	32/51 (62.7%)	7.689/0.006^a^

^a^Chi square, ^b^parametric t-test, and ^c^Mann-Whitney U-Test used as appropriate. ^d^Categorical variables with cell frequencies lower than 5 were not analysed due to requirement violations of the Chi-square tests

Parameters highlighted in bold indicate significant differences between the groups

**Table 4:** Predictors associated with good long-term outcome after undergoing mechanical thrombectomy assessed by telephone and dichotomized EQ self-care score by the EQ-5D-5L (score 1 or 2 versus greater than or equal to 3)

	Self-care score EQ-5D-5L 1 or 2 (N = 66)	Self-care score EQ-5D-5L 3–5 (N = 37)	Test statistics
Age	64.9 ± 15.2	72.6 ± 14.2	**U = 1635.000, Z = 2.847, *p* = 0.004**^**c**^
Severity indices on admission			
NIHSS on admission	12.1 ± 5.9	16.3 ± 6.4	**U = 1489.500, Z = 3.343, *p* < 0.001**^**c**^
	Median: 13	Median: 18	
NIHSS after 24 h	5.8 ± 5.7	12.9 ± 7.1	**U = 1197.500, Z = 4.302, *p* < 0.001**^**c**^
	Median: 5	Median: 11.5	
Delta NIHSS baseline to 24 h ≥ 8	23/54 (42.6%)	4/25 (16.0%)	**5.372/0.020**^**a**^
NIHSS ≤ 10 after 24 h	48/58 (82.8%)	11/26 (42.3%)	**14.052 / < 0.001**^**a**^
Thrombectomy parameters			
Thrombectomy technique			
Stentimplantation	16/66 (24.2%)	1/37 (2.7%)	**7.982/0.005**^**a**^
Risk factors			
Atrial fibrillation	23/66 (34.8%)	21/37 (56.8%)	**4.650/0.031**^**a**^
Complications			
Infection within 72 h	28/66 (42.4%)	25/37 (67.6%)	**6.000/0.014**^**a**^

^a^Chi square, ^b^parametric t-test, and ^c^Mann-Whitney U-Test used as appropriate. ^d^Categorical variables with cell frequencies lower than 5 were not analysed due to requirement violations of the Chi-square tests

Parameters highlighted in bold indicate significant differences between the groups

**Table 5:** Predictors associated with good long-term outcome after undergoing mechanical thrombectomy assessed by telephone and dichotomized EQ usual activities score by the EQ-5D-5L (score 1 or 2 versus greater than or equal to 3)

	Usual activity score EQ-5D-5L 1 or 2 (N = 50)	Usual activity score EQ-5D-5L 3–5 (N = 53)	Test statistics
Age	62.9 ± 15.8	72.2 ± 13.3	**U = 1827.000, Z = 3.314, *p* < 0.001**^**c**^
Severity indices on admission			
NIHSS on admission	11.7 ± 5.8	15.3 ± 6.4	**U = 1522.000, Z = 2.721, *p* = 0.007**^**c**^
	Median: 13	Median: 16	
NIHSS after 24 h	5.6 ± 5.9	10.6 ± 7.1	**U = 1275.000, Z = 3.547, *p* < 0.001**^**c**^
	Median: 4.5	Median: 8	
NIHSS ≤ 10 after 24 h	37/44 (84.1%)	22/40 (55.0%)	**8.482/0.004**^**a**^
Thrombectomy parameters			
Thrombectomy technique			
Stentimplantation	12/50 (24.0%)	5/53 (9.4%)	**3.961/0.047**^**a**^
Risk factors			
Hypertension	37/50 (74.0%)	50/53 (94.3%)	**8.112/0.004**^**a**^
Blood glucose level on admission	129.7 ± 56.4 mg/dl	134.2 ± 28.8 mg/dl	**U = 1564.000, Z = 2.165, *p* = 0.030**^**c**^
Statine therapy on admission	5/32 (15.6%)	19/44 (43.2%)	**6.511/0.011**^**a**^
Atrial fibrillation	16/50 (32.0%)	28/53 (52.8%)	**4.562/0.033**^**a**^
Recent therapy			

^a^Chi square, ^b^parametric t-test, and ^c^Mann-Whitney U-Test used as appropriate. ^d^Categorical variables with cell frequencies lower than 5 were not analysed due to requirement violations of the Chi-square tests

Parameters highlighted in bold indicate significant differences between the groups

**Table 6:** Predictors associated with good long-term outcome after undergoing mechanical thrombectomy assessed by telephone and dichotomized EQ pain/discomfort score by the EQ-5D-5L (score 1 or 2 versus greater than or equal to 3)

	Pain/discomfort score EQ-5D-5L 1 or 2 (N = 65)	Pain/discomfort score EQ-5D-5L 3–5 (N = 38)	Test statistics
Thrombectomy parameters			
Occluded target vessel			
M1	39/65 (60.0%)	30/38 (78.9)	**3.893/0.048**^**a**^
Risk factors			
Blood glucose level on admission	124.3 ± 27.3 mg/dl	145.6 ± 63.1 mg/dl	**U = 1458.000, Z = 2.198, *p* = 0.028**^**c**^
Former stroke	9/65 (13.8%)	13/38 (34.2%)	**5.921/0.015**^**a**^
Complications			
Intracerebral bleeding	31/65 (47.7%)	9/38 (23.7%)	**5.819/0.016**^**a**^

^a^Chi square, ^b^parametric t-test, and ^c^Mann-Whitney U-Test used as appropriate. ^d^Categorical variables with cell frequencies lower than 5 were not analysed due to requirement violations of the Chi-square tests

Parameters highlighted in bold indicate significant differences between the groups

**Table 7:** Predictors associated with good long-term outcome after undergoing mechanical thrombectomy assessed by telephone and dichotomized anxiety score by the EQ-5D-5L (score 1 or 2 versus greater than or equal to 3)

	Anxiety score EQ-5D-5L 1 or 2 (N = 55)	Anxiety score EQ-5D-5L 3–5 (N = 48)	Test statistics
*Recent therapy*			
Antidepressive therapy at telephone interview	5/55 (10.9%)	13/46 (28.3%)	4.938/0.026^a^

^a^Chi square, ^b^parametric t-test, and ^c^Mann-Whitney U-Test used as appropriate. ^d^Categorical variables with cell frequencies lower than 5 were not analysed due to requirement violations of the Chi-square tests

Parameters highlighted in bold indicate significant differences between the groups

After testing for multicollinearity, multivariate logistic regression analysis was performed for each dimension. Age less than 70 years (OR 4.62, CI 2.43–8.80, *p* < 0.001) and NIHSS score at 24 h less than or equal to 10 (OR 4.08, CI 1.97–8.44, *p* < 0.001) emerged as predictors of a more favourable outcome (activity score 1 or 2) for the dimension activity of the EQ-5D-5L. For the self-care dimension, predictors of a more favourable outcome (activity score 1 or 2) were also age less than 70 years (OR 3.33, CI 1.78–6.21, *p* < 0.001), a delta NIHSS score between admission and 24 h greater than or equal to 8 (OR 1.98, CI 1.04–3.78, *p* = 0.038), and a 24-h NIHSS score less than or equal to 10 (OR 3.41, CI 1.80–6.44, *p* < 0.001). For the usual activity dimension, in addition to age below 70 years (OR 4.51, CI 1.99–10.22, *p* < 0.001) and a 24-h NIHSS score of 10 or less (OR 6.80, CI 2.57–18.00, *p* < 0.001), a lower glucose level on admission (OR 1.01, CI 1.00–1.02, *p* = 0.042) was shown to be a predictor of better long-term activity (score 1 or 2). In contrast to the first three dimensions, other predictors of a favourable outcome were found for pain/discomfort (lower glucose level on admission, OR 1.01, CI 1.00–1.01, *p* = 0.034; negative predictor: former stroke, OR 0.56, CI 0.33–0.97, *p* = 0.039) and for anxiety (no predictors during the inpatient stay, but antidepressant therapy in the long-term as a negative predictor: OR 0.27, CI 0.15–0.49, *p* < 0.001).

## Discussion

The present study aimed at identifying predictors for a good long-term outcome in patients receiving EVT. In addition, the relationship between favourable long-term outcome and HRQoL was analysed. 694 AIS patients receiving EVT between 01/2017 and 12/2020 were retrospectively assessed. 138 patients performed a telephone survey with a mean follow-up interval of 2.2 ± 1.2 years (median: 2.1 years) and participated in the long-term outcome analysis.

Independent predictors for a good long-term outcome were age below 70, lower NIHSS on admission, NIHSS at 24 h post EVT ≤ 10, and complete recanalisation (mTICI 3) after EVT. Occurrence of infections requiring treatment within the first 72 h was a negative predictor for good long-term outcome. The dimensions mobility, self-care, and usual activities of HRQoL were positively correlated to favourable long-term outcome.

The novelty of this study is the comprehensiveness of the analysis of predictors of long-term outcome (beyond day 90), its relation to HRQoL, as well as predictors of intrahospital mortality and recanalisation success in a large European cohort.

### Long-term outcome after EVT

In addition to comprehensiveness, an important difference of our analysis as compared to previously published studies is the prolonged follow-up interval after stroke. Most published studies evaluated clinical outcome using mRS at day 90 [3, 10, 12, 26, 28, 31–33, 37, 38, 46, 47, 50–52]. Only a few studies assessed the clinical outcome beyond 90 days after the index event [5, 14, 16, 36, 53]. Beyeler et al. [5] analysed the long-term functional outcome (mRS) and health-related quality of life (EQ-5D-3L) according to the baseline Alberta Stroke Program Early Computed Tomography Score (ASPECTS) using follow-up telephone interviews with a median of 3.67 years. A higher eTICI reperfusion grade after EVT was shown to be a predictor of better long-term functional outcome (mRS 0–3) and quality of life in the low ASPECTS group (0–5) with one third of patients achieving a favourable outcome. The authors were also able to show that the poor long-term quality of life in patients with low ASPECTS was mainly the result of constraints affecting mobility, self-care, and usual activities [5]. Thus an impairment of the same dimensions was reported for the EQ-5D-5L. We showed in the present study, that in these first three dimensions, with predominantly motor, functional and activity-based dimensions, predictors of a good long-term outcome were age below 70 years and a NIHSS score at 24 h less than or equal to 10. Interestingly, patients with initially elevated blood glucose levels had poorer usual activity scores. This finding correlates with the results of a recent meta-analysis, showing that acute stroke patients with elevated blood glucose levels who received systemic thrombolytic therapy had a worse outcome as measured by mRS at 90 days and a higher rate of symptomatic intracerebral haemorrhage [49]. Possible mechanisms for such detrimental effects of hyperglycaemia have been discussed and include exacerbation of pathophysiological mechanisms such as thrombo-inflammation known to negatively impact infarct evolution final outcome [9, 11, 40, 41].

In an observational, nationwide registry of consecutive Chinese patients, 208 of 807 patients (25.8%) reached favourable outcome (mRS 0–2) after five years, whereas 48.2% of the patients were dead after five years [16]. Younger age, lower mRS at 90 days, and absence of stroke recurrence were associated with favourable outcome at five years. In our study, 134 patients died during hospitalisation and a total of 169 patients died till the end of the follow-up period. Despite the rate of missing participants in the telephone interview, 169 of the 694 patients in the entire collective (24.4%) died within 2.1 years of the stroke event.

Our data demonstrate an age below 70 years, lower NIHSS on admission, NIHSS score at 24 h post EVT ≤ 10, and complete recanalisation mTICI 3 after EVT as independent predictors of favourable outcome with a median time of 2.1 years after stroke. Younger age has already been identified as a relevant influencing factor in various publications [10, 16, 31, 33, 51, 52]. However, it is important to emphasize that older patients do not have an unfavourable prognosis per se. In a study with nonagenarians (patients aged ≥ 90 years) who underwent EVT, 28.9% achieved a favourable clinical outcome after 90 days (mRS 0–2) [46]. Severity on admission measured by NIHSS is a relevant predictor of outcome in some analyses [3, 10, 12, 26, 33, 52, 53]. The NIHSS score at 24 h post intervention with a threshold of ≤ 8 has also been described as the best surrogate marker for long-term functional outcome after thrombectomy of the anterior circulation. Another recent study evaluated the absolute 24-h NIHSS score ≤ 10 and a delta NIHSS score ≥ 8 between baseline and 24 h to be associated with good functional outcome after 3 months [50]. In our data, a delta NIHSS ≥ 8 was also shown to be a predictor of good long-term outcome, but the regression model showed a slightly reduced statistical power overall.

Furthermore, the outcome is highly dependent on the time course of EVT. Shorter time from stroke onset to reperfusion and successful recanalisation are important prognostic parameters [10, 37, 38, 47, 52]. Fuhrer et al. [14] described a decline in mRS after one year compared to mRS at day 90 in patients with right hemispheric infarctions. In our cohort, even in a subgroup of patients who were discharged alive (mRS at discharge ≤ 5), there was no significant dependence on the infarction side in terms of long-term outcome.

### The impact on HRQoL

Our study shows an association between motor function impairment, as measured by the mRS, and HRQoL, especially in the specific dimensions of activity, self-care and habitual activities. Patients after EVT with older age and severe deficit after EVT show an increased risk of long-term HRQoL impairment in addition to mRS impairment.

Living with the consequences of a stroke can affect the physical and mental health of patients, resulting in a reduced health-related quality of life (HRQoL) [30]. A long-term survey of HRQoL in stroke patients is relevant for the development of possible strategies for improvement. HRQoL outcomes were shown to be significantly influenced by different factors at different time points after stroke: While bodily functions, activities and participation (mainly personality functions and recreation and leisure) showed significance on HRQoL within 3 months after stroke, environmental factors showed a greater influence on HRQoL at 1 year [2]. Except for those with excellent functional recovery, even young patients after ischaemic stroke have poorer long-term HRQOL [44]. Despite significant improvement in motor scales such as the NIHSS and mRS after acute stroke during hospitalisation and the following 12 months, HRQoL remained below that of the age-matched general population at 12 months, but was still unexpectedly high given the prevalence of permanent disability in up to 30% of patients [25]. The temporal changes in the course of stroke and the various influencing factors highlight the importance of assessing HRQoL beyond a 3-month follow-up, especially in patients with severe stroke such as those who have undergone EVT, as improvements often only become apparent in the long term. In this context, recent data analysing predictors of excellent quality of life at 2.5 years after stroke receiving thrombolytic therapy are quite interesting. These included age < 73 years, a low NIHSS score after IVT and the absence of hypertension. Quality of life was assessed in all dimensions with a mean score of 1 and a mean EQ-VAS of 70, showing the good general health of this stroke population [43]. In general, dimension-specific factors influencing HRQoL were observed in Korean stroke patients [27]: older age was associated with mobility problems, self-care problems increased with age, and depression. Problems with usual activity increased with increasing age, low income, lack of economic activity and depression. Low income also influenced problems with pain/discomfort. The total EQ-5D index score was lower in stroke survivors with older age, hypertension, diabetes mellitus, and lack of regular exercise. Even five years after stroke, four out of five affected individuals have HRQoL impairments related to at least one of the five dimensions of the EQ-5D-3L, with pain/discomfort being the most commonly reported HRQoL impairment. Older age and longer hospital stay were associated with HRQoL impairment [45]. Understanding how HRQoL is affected over the long-term and identifying predictors of high risk for low HRQoL can help to identify patients who may benefit from special attention and psychological support. Our results show that there is a relationship between motor-functional impairment measured by the mRS and HRQoL, especially in the specific dimensions of activity, self-care, and usual acitivities. Patients after EVT with higher age and severe deficit after EVT show an increased risk of long-term impairment in HRQoL in addition to impairment in mRS.

### Study limitations

A study immanent limitation represents the selection bias regarding good outcome. The reason for this is twofold: Written informed consent was required for participation in the telephone survey. Patients who declined to participate or did not return a signed consent form were not included in the analysis—producing a bias towards a population of responsive and health-oriented patients. On the other hand, QoL was measured by a self-assessment via telephone interview requiring the patient or its relative to be able to talk on the phone. Patients with poor health conditions living in a nursing home or getting professional nursing care at home are presumably underrepresented. Design and conductance of the present study type is prone for a selection bias towards healthier patients which is immanent for this type of studies and independent of the presented one. This is also supported by the analysis that the telephone survey sample was younger and had a lower mRS at discharge than the general population. Statistically, the difference in mRS was mainly due to the higher proportion of patients who died at discharge in the overall population compared to the proportion of patients who died during long-term follow-up. However, due to the study design and the data protection regulation, it was much more difficult to collect data from relatives of patients who had already died or were living in a nursing home than it was to collect data from patients who were able to participate in everyday life independently.

## Conclusion

Our data enhance previous findings of predictors for good and poor outcome following EVT. Independent predictors for a good long-term outcome were age below 70, lower NIHSS on admission, NIHSS at 24 h post EVT < 10, and complete recanalisation (mTICI 3) after EVT.

Of particular relevance and novelty is the finding that patients with ischaemic stroke and EVT have impaired HRQoL that is still detectable more than 2 years after the stroke event. Here, dimension-specific impairments in activity, self-care and usual activities are related to functional impairment as measured by mRS. Older age and greater severity after EVT are significant predictors of worse dimension-specific HRQoL in these domains, whereas high blood glucose levels on admission are an additional predictor of worse outcome in the domain of usual activities. We believe that knowledge of HRQoL impairment over the long term is highly relevant for early attention in rehabilitation and targeted training of deficits. In addition, psychological monitoring could also be beneficial to improve HRQoL in the long term. Further studies will be very useful in this regard.

## Data Availability

The data that support the findings of this study are available from the corresponding author upon reasonable request.

## Abbreviations

AIS: Acute ischemic stroke
ASPECTS: Alberta Stroke Program Early Computed Tomography Score
EVT: Endovascular thrombectomy
IV-rtPA: Intravenous recombinant tissue plasminogen activator
LTO: Long-term outcome
LVO: Large vessel occlusion
MRI: Magnetic resonance imaging
mRS: Modified Rankin Scale
mTICI: Modified treatment in cerebral infarction
NIHSS: National Institutes of Health Stroke Scale
HRQoL: Health-related quality of life

## Code availability

Statistical analysis of the data was carried out using the Statistical Package for the Social Sciences (SPSS) version 25 (IBM, 2018).
